# Lake SkyWater—A Portable Buoy for Measuring Water-Leaving Radiance in Lakes Under Optimal Geometric Conditions

**DOI:** 10.3390/s25051525

**Published:** 2025-02-28

**Authors:** Arthur Coqué, Guillaume Morin, Tiphaine Peroux, Jean-Michel Martinez, Thierry Tormos

**Affiliations:** 1 Pôle R&D ECLA, 13100 Aix-en-Provence, France; tiphaine.peroux@inrae.fr (T.P.); thierry.tormos@ofb.gouv.fr (T.T.); 2 Aix Marseille University, INRAE, RECOVER, 13100 Aix-en-Provence, France; 3 Géosciences Environnement Toulouse (GET), University of Toulouse, CNRS, IRD, CNES, 31400 Toulouse, France; jean-michel.martinez@ird.fr; 4 Magellium, 31520 Ramonville Saint-Agne, France; guillaume.morin@magellium.fr; 5 Service EcoAqua, DRAS, OFB, 13100 Aix-en-Provence, France

**Keywords:** radiometric buoy, skylight-blocked approach (SBA), remote sensing reflectance, water-leaving radiance, field radiometry, water colour, water quality monitoring

## Abstract

This study introduces Lake SkyWater (LSW), a novel radiometric buoy designed for the reliable measurement of remote sensing reflectance (R_rs_) in lakes using the Skylight-Blocked Approach (SBA). LSW addresses key challenges in “on-water” field radiometry owing to its motorised rotating system, which maintains the radiance sensor in optimal geometrical conditions (i.e., facing the sun). Our device is easy to transport and deploy and can be controlled with a smartphone over Wi-Fi. Its modular design, which uses standard components and custom 3D-printed parts, facilitates customisation. A field experiment demonstrated excellent performance in the visible spectrum (400–700 nm) and no significant differences compared with handheld SBA measurements when measuring R_rs_ (coefficient of determination > 0.99 and general accuracy (median symmetric accuracy) of ~2.43%). Areas for potential improvement were identified, such as refinement of orientation control and addressing the occasional rotation of the float. Nonetheless, LSW shortens the acquisition time, reduces the risk of fore-optics contamination, and ensures that the measurements are conducted under optimal geometric conditions. In conclusion, LSW is a promising instrument for the operational collection of high-quality R_rs_ spectra in lakes, which is important for advancing both research and monitoring applications in aquatic remote sensing.

## 1. Introduction

Optical remote sensing has emerged as a powerful tool for monitoring water quality on a global scale [1,2,3] and for achieving a better understanding of various aquatic ecosystems. It uses data obtained from space- or air-borne sensors to obtain near real-time information regarding the constituents of the water column across vast and potentially inaccessible areas.

Water colour remote sensing relies on the interaction between light and water constituents, typically in the visible (400–700 nm) and near-IR (700–1000 nm) parts of the spectrum. When sunlight penetrates a water body, its spectral properties are modified based on the absorption and scattering characteristics of water, depending on its dissolved and particulate constituents and their concentrations. Part of this altered light is scattered back to the surface and potentially captured by optical sensors and is called the water-leaving radiance (L_w_, in W m^−2^ sr^−1^ nm^−1^). For comparison with satellite imagery, researchers mostly use the remote sensing reflectance (R_rs_, in sr^−1^), defined as the ratio of L_w_ to the downwelling irradiance at the water surface (E_s_, in W m^−2^ nm^−1^). R_rs_ contains information about the water body and its constituents, making it a critical parameter for assessing water quality and other aquatic characteristics. For instance, it is used to derive chlorophyll-a and CDOM concentrations or turbidity, but also for detecting submerged macrophytes (e.g., seagrass and kelp beds) or bottom depths in shallow waters [4,5,6].

In situ radiometric measurements, if they are valuable as such for conducting environmental studies [7], are also key to the development and validation of water colour algorithms by linking space-based observations to ground-based knowledge. High-quality in situ measurements are also required for the system’s vicarious calibration of spaceborne sensors. For instance, a community work [8] recommends that in situ reference measurements in oligotrophic and mesotrophic areas should reach an uncertainty lower than 3–4% for the L_w_ in the blue–green spectral regions and 5% in the red.

Despite the importance of R_rs_ measurements, too little data have been collected in the field. Most of the difficulty arises from the costs of materials and campaigns. Also, the technicity required to manipulate radiometric devices does not guarantee the success of a campaign and the quality of the measured data.

Various methods exist for measuring R_rs_ in the field [9,10,11], but the Skylight-Blocked Approach (SBA) [12,13,14] is noteworthy for its ability to directly measure L_w_ instead of deriving it from the upwelling radiance. This protocol allows high-quality measurements, even in complex environments (e.g., in stratified or shallow waters, or in the presence of a scattered cloud cover). It consists of measuring the upwelling radiance close to the water surface using a nadir-view radiometer equipped with a shield screening skylight at its open end, thus preventing sky- and sun-glint from entering the sensor FOV. The signal recorded by the radiance sensor represents the actual radiance emerging underneath the water surface, L_w_. It is expected not to be polluted by the signal coming from the light reflecting at the water surface, such as for a standard “above-water” protocol [15]. Concomitant and collocated measurements of E_s_ allow for the derivation of the R_rs_. Manually operated SBA methods (used in [16,17,18]) require constant attention to maintain the radiance sensor immersed at the right depth and in the correct position with respect to the sun and deployment platform during the entire acquisition sequence. If standard protocols [19] recommend long-lasting sequences of acquisition (typically 5–10 min), rough water conditions often make this exercise challenging and wearing and fully monopolises an operator dedicated to this measurement. Knowing that, a few authors [14,20,21] have designed floating systems implementing the SBA method, herein called “radiometric buoys”. These radiometric buoys allow for easier deployment and enable measurements to be taken at a reasonable distance from the research vessel (typically 10–20 m), in a location where the aquatic light field will not be affected by the presence of the vessel. However, optimal geometric conditions, which are crucial for obtaining accurate SBA measurements, are not necessarily ensured. For instance, radiometric buoys can rotate freely under the effect of winds or waves, causing the radiance sensor to measure the L_w_ of a water volume in the direct sun shadow of the buoy, thus biasing L_w_ measurements [22]. These floating devices are also subject to wave-induced tilt, which moves the sensors away from the nadir view. Changes in the sun-relative zenith angle of the irradiance sensor (i.e., the difference between the sun zenith angle and the zenith angle of the radiometer) can cause a large bias, particularly if the radiometer is tilted towards (or away from) the sun [23]. The tilt issue is typically addressed by measuring the tilt of the instruments with onboard inclinometers and later discarding impaired radiometric measurements in the post-processing step. However, there is currently no measuring system that ensures the correct sun-relative azimuth of the radiance sensor throughout the entire acquisition sequence. It relies on intensive manipulation control during the whole acquisition (for handheld SBA), or on manual quality control during the post-processing step (for all SBA implementations), impeding the assurance of de facto accurate and consistent measurements in the field.

The present study aims to offer a novel solution, called Lake SkyWater (LSW), to reliably and easily acquire R_rs_ in lakes. Its qualities, reasonable cost, and ease of construction would enable the water colour community to better and more easily document the optical properties of water worldwide. In this article, we describe the LSW design, which implements the SBA scheme and ensures that the measurements are performed under optimal geometric conditions, evaluate its performance in the field, and discuss its advantages.

## 2. Lake SkyWater Design

### 2.1. General Idea and Naming Conventions

Radiometric buoys need to be easy to handle and transport, sturdy enough to hold the sensors, and small enough to minimise the impact on the ambient light field. We chose to keep the classic design of SBA buoys [19] consisting of two radiometers in the nadir view on opposite sides of a float: one (the irradiance sensor) looks upward and measures E_s_, and the other (the radiance sensor) faces downward and measures L_w_.

**Figure 1:**
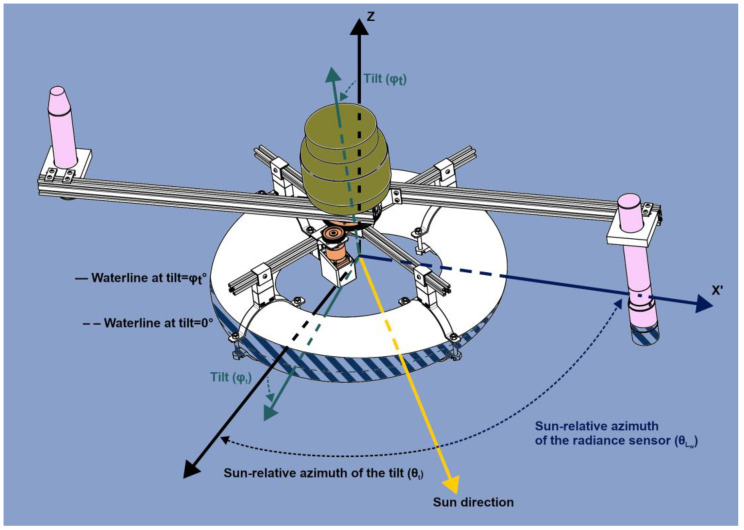
Lake SkyWater design and geometry. Sun-relative azimuths of the tilt and the radiance sensor (θ_t_ and θ_Lw_, respectively). The waterline at a given tilt (φ_t_) is a plain line, and the submerged part of the device is hatched. The waterline if φ_t_ were 0° is materialised as a dashed line.

The key addition of LSW is its motorised solution, which ensures that the radiance sensor is placed in optimal geometrical conditions with respect to the sun and buoy during the entire measurement sequence. For this purpose, LSW is composed of four sections (Figure 1) detailed hereafter: the structure (in white), the rotating system (in orange), the radiometric system (in pink), and the embedded system (inside the green container). We designed the custom parts using Onshape and printed them using the Netpune 4 entry-level 3D printer (Elegoo, 518109 Shenzhen, China). These 3D models as well as the code for controlling the device are freely available on GitHub at https://github.com/inrae/Lake-SkyWater (v1.0; accessed on 24 February 2025).

To facilitate the understanding of the results, we propose a naming convention for all the variables that we use hereafter.

Let X, Y, and Z be a referential centred on the LSW centroid, whose three axes are fixed: X points eastwards, Y northwards, and Z is orthogonal to the geoid (i.e., the water surface) and points upwards.

Let X’, Y’, and Z’ be a referential centred on LSW centroid, whose three axes rotate with the two arms to which the sensors are attached (X’ points in the direction of the radiance sensor).

When LSW is at rest (i.e., without any tilt): $\hat{z}={\hat{z}}^{\prime }$, with $\hat{z}={\left[\begin{array}{ccc} 0 & 0 & 1 \end{array}\right]}^{T}$.

In the presence of waves, LSW rotates from its reference position such that ${\hat{z}}^{\prime }=R\cdot \hat{z}={\left[\begin{array}{ccc} x_{z} & y_{z} & z_{z} \end{array}\right]}^{T}$, where *R* is a general 3D rotation matrix. By converting Cartesian coordinates to spherical coordinates, we obtain the following:

- $r=\sqrt{x_{z}^{2}+y_{z}^{2}+z_{z}^{2}}$, the radial distance of ẑ′ in the (X, Y, Z) referential;
- $\Theta_{t}=atan2\left(\frac{x_{z}}{y_{z}}\right)$, the azimuth of ẑ’ in the (X, Y, Z) referential, defined on the interval [0°, 360°). The sun-relative azimuth angle of the tilt θ_t_ (defined on (−180°, 180°]) is then derived: $\theta_{t}\equiv \Theta_{t}-SAA$, where SAA is the solar azimuth angle (defined on [0°, 360°));
- $\varphi_{t}=arccos\left(\frac{z_{z}}{r}\right)$, the polar angle of ẑ in the (X, Y, Z) referential, i.e., the tilt “amplitude”, defined on [0°, 180°].

The final angle of importance is θ_Lw_, the sun-relative azimuth angle of the radiance sensor. When LSW rotates (either because of waves or its rotating system), ${\hat{x}}^{\prime }=R\cdot \hat{x}={\left[\begin{array}{ccc} x_{x} & y_{x} & z_{x} \end{array}\right]}^{T}$, where *R* is a general 3D rotation matrix. In the same way as for the tilt, we obtain $\Theta_{Lw}=atan2\left(\frac{y_{x}}{x_{x}}\right)$, and thus, $\theta_{Lw}\equiv \Theta_{Lw}-SAA$.

These three angles, along with their names and value ranges, are listed in Table 1.

### 2.2. The Overall Structure

The structure of LSW (see Figure 1) is composed of a float, a main frame, a small (3.6 L) waterproof container, and 3D printed parts. The float is a front tractor inner tube (major diameter of 50 cm and minor diameter of 14 cm). The frame is made of black anodised aluminium profiles (Systéal, 77185 Lognes, France). Black anodising was applied to make it more resistant and less reflective in water owing to its matte finish. It is composed of two parts connected by a slewing ring (see Section 2.2). The first axis is fixed and attached to the floating body with 3D printed clamps, while the second (consisting of two 74 cm arms to which the radiometers are attached) can rotate, allowing the correct orientation of the radiometer measuring L_w_. The waterproof container is used to safely store all the electronics: the embedded system and power supply (see Section 2.4). It is attached to the rotating part or the structure using 3D printed clips, in the centre of the float. All 3D printed parts (clamps, clips, wedges, etc.) were made of PETG, which is ideal for functional 3D printing because it is UV- and water-resistant, stronger than PLA, and easier to print than ABS. These custom elements allow LSW to be adaptable to different hardware (sensors, floats, etc.) and protocols (e.g., by modifying the distance between the radiometers and the water surface).

### 2.3. The Rotating System

A rotating system has been added compared to the classic design of SBA radiometric buoys [19], enabling the radiometer measuring L_w_ to always be placed under optimal geometrical conditions, i.e., facing the sun so that it does not measure water in the direct shadow of the structure. The rotation is made possible by the use of a slewing ring driven by a stepper motor. We chose the Iglide^®^ PRT-04 slewing ring (igus GmbH, 51147 Köln, Germany), an on-the-shelf product with high performance, robust design, and moderate price. The motor (STEPPERONLINE, 211100 Nanjing, China) is a NEMA 17 stepper equipped with a high-precision planetary gearbox. This low-cost option has good accuracy (maximal backlash of 50 arc minutes) and provides a significant torque (maximal permissible torque of 10 N m), which is required for easily rotating the moving chassis and the attached radiometers.

### 2.4. The Radiometric System

Two synchronised radiometers are required to implement the SBA scheme. One for measuring L_w_ and the other to measure the surface irradiance E_s_. A cone- or cylinder-shaped apparatus attached to the radiance sensor (hereinafter referred to as “screen”) is also needed to block sky- and sun-light reflection at the water surface, allowing the direct measurement of L_w_. In this study, we used two RAMSES G2 VIS hyperspectral radiometers (TriOS Mess- und Datentechnik GmbH, 26180 Rastede, Germany), which are widely used in the water colour community [24]. These two sensors operate in the 320–950 nm wavelength range, with a spectral sampling of ~3.3 nm and an adjustable integration time of 4 ms to 8 s. The radiance sensor has an FOV of 7°. They are lightweight (<1 kg) and have a low power consumption (~1 W), making them suitable for portable and autonomous use. Similar to the other 3D printed parts, the conical screen was made of matte black PETG. It measures ~9.5 cm in height and 5.8 cm in width and can be screwed to the radiometer using a custom-made adapter (collar). In this study, the immersion depth of the screen was ~3.7 cm. We calculated (considering the dimensions of the device) that this depth allowed the open end of the screen to remain below the water surface as long as the buoy was not tilted by more than 5°. The smaller the screen and its immersion depth, the better it is for self-shading, i.e., the contamination caused by the shadow of the radiance sensor and its attached screen in water (see [25] for its evaluation).

### 2.5. The Electronic System

All electronics, i.e., the embedded system and power supply, are safely housed in a waterproof container placed in the centre of the buoy.

The embedded system is made of open-source components, and is composed of a Raspberry Pi 3 Model B+ (RPi; Raspberry Pi Ltd, CB4 0AB Cambridge, UK) and Tinkerforge modules (Tinkerforge GmbH, 33758 Schloß Holte-Stukenbrock, Germany) for I/O and add-on sensors. The RPi serves as a local controller and data logger. It controls the radiometers and the stepper motor and allows for wireless communication with the end user’s device (e.g., a laptop, a smartphone, etc.) via a self-hosted Wi-Fi network. All measured data are stored on the same SD card as the operating system (64-bit Raspberry Pi OS). Tinkerforge is a flexible and affordable system of building blocks that is typically used for prototypes and small batch production. These blocks can be divided into two categories: Bricks and Bricklets. Bricks are single-task stackable 44 cm PCBs. Bricklets can be connected to Bricks and allow for the addition of sensors (here, an inertial measurement unit (IMU) and a GNSS receiver) and IO interfaces (here, RS485/Modbus RTU for communicating with the two radiometers and the Silent Stepper Brick for controlling LSW stepper motor) to the system. The IMU + GNSS receiver combination is required for positioning the radiance sensor (see Section 2.5), as well as for monitoring the location of the buoy. The geolocation of LSW is provided by the Tinkerforge GPS Bricklet 2.0. It is used to record a time series of latitude, longitude, and altitude at each sampling station, eventually monitoring the drift of the buoy during a measurement sequence, and to set the time of the system, avoiding a battery-backed real-time clock. This Bricklet is equipped with a FireFly X1 (GlobalTop Technology Inc., 741 Tainan, Taiwan), which is a compact low-powered multi GNSS module that can provide a positioning accuracy of up to 1.8 m CEP (circular error probability), and is connected by a U.FL connector to an external GPS antenna attached on top of the waterproof container. The absolute orientation of LSW is provided at any time by the Tinkerforge IMU Bricklet 3.0. It is used in combination with the location of the buoy and the current time to compute the angles illustrated in Figure 1: θ_Lw_, θ_t_, and φ_t_.

The power source of LSW—a 24V 2Ah NiMh battery—is connected to (i) the Tinkerforge Step-Down Power Supply, powering the stepper motor through the Silent Stepper Brick, (ii) the Tinkerforge HAT Brick, powering the RPi and all the Bricklets, and (iii) the two radiometers. It allows <20 h of measurements or ~1 h of usage of the stepper motor “at full speed” (see Table S1 for the list of all the electronics and the power consumption of each item). Given that we need ~5 min per sampling station, i.e., ~75 R_rs_ spectra, this means we can measure up to 10 stations per battery.

### 2.6. The Acquisition Protocol and Output Data

The standard scenario used for field measurements includes the following stages: (i) switching on LSW, (ii) connecting to it through SSH, and (iii) starting the acquisition sequence using the provided command line interface (CLI). At the end of the measurements, the raw spectra are calibrated (i.e., corrected for the dark signal and non-linear response, using the relevant calibration coefficients), and summary graphs are generated. These can be used to check the data quickly. The measured data (Table 2) can then be downloaded for further processing and visualisation.

When the acquisition sequence starts, two scripts are launched in parallel; the first controls the rotating system (see Section 2.2) and the other controls the radiometric system (see Section 2.3).

The pseudo-code of the orientation algorithm is provided below. Please, confer to Section 2.1 and Figure 1 for all the above-mentioned angles and their definition.

- Every minute: compute the solar azimuth angle (SAA) from the current time and location of the buoy using pvlib (NREL SPA algorithm [24,25]). Time and location are provided by the GPS Bricklet;
- Every 400 ms (the default value):
  1. Compute the sun-relative azimuth of the radiance sensor (θ_Lw_) from both the SAA and the absolute orientation of the buoy (r, provided by the IMU);
  2. As we want the radiance sensor to face the sun, θ_Lw_ must be set to 0°. Therefore:
    - If $\left|\theta_{Lw}\right|>5{}^{\circ}$: compute the number of steps (n_steps_) the stepper motor should run so that $\theta_{Lw}=5{}^{\circ}$. $n_{steps}=-int\left(\theta_{Lw}/d\theta \right)$, where int is the Python 3 int() function and dθ is the step angle (a constant of the stepper motor + gearbox ensemble, here equal to 0.036°);
    - Else: do nothing.

The script controlling the two radiometers allows for the acquisition of a given number of L_w_ and E_s_ spectra. Both radiometers are triggered synchronously.

The logged data are summarised in Table 2. The sun-relative azimuth of the radiance sensor θ_Lw_ and LSW tilt (both its amplitude φ_t_ and direction θ_t_) are not saved as they can easily be derived from the absolute orientation of the buoy r, its location (latitude and longitude), and the time_1–2_.

## 3. Prototype Testing

### 3.1. Study Site and Experimental Setup

**Figure 2:**
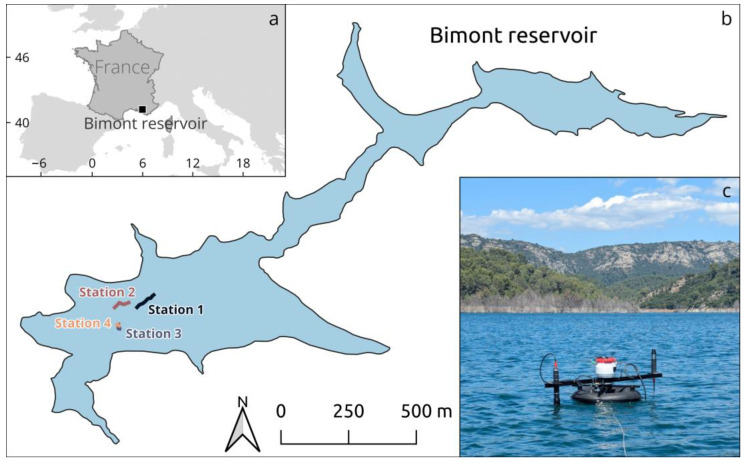
Study area (Bimont reservoir). (**a**) Location of Bimont reservoir; (**b**) Location of the sampling stations (+drift of the boat/buoy during measurements). (**c**) Lake SkyWater deployed on the reservoir.

LSW performance assessment was conducted in early July 2024 in Bimont reservoir (Figure 2), located in southeastern France. This oligotrophic impoundment of 14 × 106 m^3^ and 74 ha has a maximal depth of 60 m at its normal operating level and is mainly used for securing the water supply to Aix-en-Provence and Marseille urban areas.

This evaluation consisted of comparing collocated measurements from two systems: LSW and a handheld SBA protocol, considered in this study as the reference.

The handheld SBA implementation also required two hyperspectral radiometers. We used equivalent radiometers to those mounted on LSW (“non-G2” TriOS RAMSES; see Figure S1 for prior comparison of the four radiometers using a TriOS FieldCAL). We attached the exact same 3D-printed airtight cylinder-shaped screen to the downward-looking sensor measuring L_w_. This radiometer was hung at the end of a 4 m telescopic boom oriented away from the ship in the direction of the sun. Particular attention was paid to ensure that the immersion depth of the screen was approximately the same (~3.7 cm) as for LSW. The irradiance sensor measuring E_s_ was onboard our boat and attached to a pole equipped with a bubble level. The two radiometers were connected to a TriOS IPS 104 interface and power supply unit and were controlled using a rugged computer.

A total of four measurement sequences (i.e., stations) were obtained (Figure 2b), from 10 h28′33″ to 14 h07′28″ UTC. Radiometers were deployed “side by side” in the field—LSW was kept over ~10–20 m away from the ship—and radiometric data were collected almost simultaneously (<2 s timespan between the two systems). A total of 75 spectra (~5 min) were acquired at each station, as well as “geometric” ancillary data: the absolute orientation of the buoy and its location. Measurements were performed with the solar zenith angle (SZA) varying between 25° and 35° and under clear sky conditions for all stations except the last, where rapidly moving cirrus clouds were present. The water surface remained calm throughout the field experiment.

### 3.2. Data Acquisition and Processing

The acquisition and preprocessing workflow for LSW is detailed in Section 2.6. For the handheld SBA, data were synchronously acquired using TriOS MDSA_XE 8.9.2 software (with measurements triggered every 4 s) and saved in a database (both raw and calibrated spectra). Calibrated radiometric measurements were then exported as CSV files for analysis and comparison with LSW data.

Before comparing the measurements from the two systems (~75 spectra per station), the calibrated ~4 s and ~3.3 nm resolution spectra were first interpolated onto a common time coordinate and then resampled between 320 and 950 nm with a 1 nm step. This resulted in a time series of instantaneous spectral L_w_(λ,t) and E_s_(λ,t) for both LSW and the handheld SBA, where *λ* is the wavelength and *t* is the observation time. The ratio of L_w_(t) to E_s_(t) is the instantaneous spectral remote sensing reflectance, R_rs_(λ,t): $R_{rs}\left(\lambda ,t\right)=L_{w}\left(\lambda ,t\right)/E_{s}\left(\lambda ,t\right)$. For simplicity, the wavelength and time dependencies are implicit for the rest of this study. Protocols are noted in superscripts, e.g., $R_{rs}^{LSW}$ and $R_{rs}^{HH}$ for R_rs_ measured using LSW and the handheld SBA, respectively. We also computed $R_{rs}^{LSW*}=L_{w}^{LSW}/E_{s}^{HH}$ so as not to be affected by calibration differences between the two irradiance sensors (see Section 4.2 and Figure S1 for further detail).

We excluded from the comparison the spectra acquired during periods showing “abnormal geometrical events” recorded by the IMU (e.g., high tilt induced by sensor blockage in the mooring rope), or when the buoy was still in the approach phase (i.e., when measurements started before the radiance sensor was in a stable position, facing the sun) (see Section 4.1).

SBA measurements are subject to errors due to instrument self-shading, which is a function of the water optical properties, SZA, and size of the radiance sensor and its attached screen. We assumed that the shading would be the same for both systems because (i) we performed synchronous collocated measurements, which implies that both the illumination condition and the water optical properties were the same for the two systems, and (ii) we used the same hardware (radiometers and screens) under the same conditions (immersion depth). We did not implement the self-shading correction recommended in [22] because we only wanted to assess the equivalence of the two systems for R_rs_ measurements.

### 3.3. Performance Metrics

The coefficient of variation (CV) was used to measure the dispersion of measurements obtained using each approach. It is defined as the ratio of the standard deviation (σ) to the mean (μ): ${CV}_{method}=\sigma_{method}/\mu_{method}$, where method refers either to LSW or the handheld SBA.

Median symmetric accuracy (MdSA) and symmetric signed percentage bias (SSPB) were used to assess the overall error and bias in the measured spectra, respectively, as recommended in [26], and are defined as follows:

(1) $$MdSA=100\times \left(10^{Y}-1\right)[\%],\;\mathrm{where}\;Y=Median\left(\left|{log}_{10}\left(R_{rs}^{LSW}/R_{rs}^{HH}\right)\right|\right)$$

(2) $$SSPB=100\times sgn\left(Z\right)\left(10^{\left|Z\right|}-1\right)[\%],\;\mathrm{where}\;Z=Median\left({log}_{10}\left(R_{rs}^{LSW}/R_{rs}^{HH}\right)\right)$$

where sgn is the sign function, and Median is the median operator. These metrics, expressed in %, are easy to interpret, robust to outliers, and symmetrically and equally penalise errors of the same order. These metrics are recommended in recent large-scale studies, such as ACIX-Aqua [27], and more broadly in water colour remote sensing, as R_rs_ values typically span several orders of magnitude depending on the water type. In addition to these two metrics, we also computed the coefficient of determination (R^2^), root mean square error (RMSE), mean absolute percentage error (MAPE), and mean percentage error (MPE), to facilitate comparisons with previous intercomparison of in situ radiometric measurements [24,28,29]. Simple linear regression slopes and intercepts were calculated with SciPy [30] using the non-linear least-squares Levenberg–Marquadt algorithm [31].

## 4. Results

### 4.1. LSW Measurements

**Figure 3:**
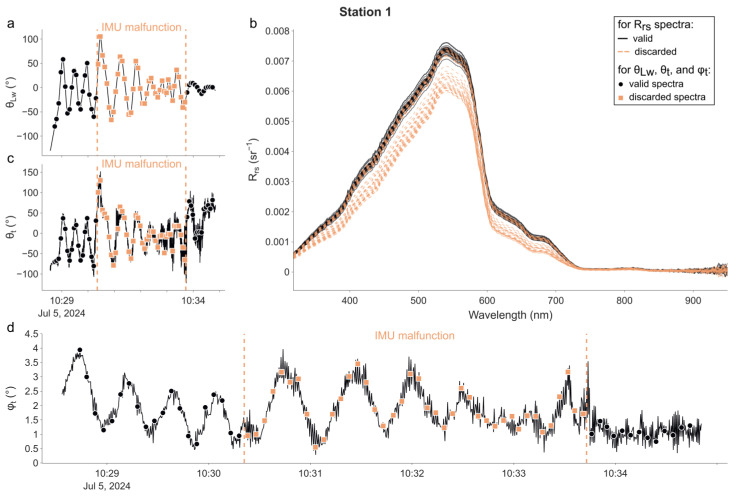
LSW measurements at the 1st station. (**a**) Sun-relative azimuth of the radiance sensor θ_Lw_; (**b**) R_rs_ spectra; (**c**) sun-relative azimuth of the tilt θ_t_; (**d**) tilt of the buoy φ_t_. The markers in panels (**a**,**c**,**d**) indicate the sampling time of all radiometric measurements. The period delimited by the two vertical dotted lines (**a**,**c**,**d**) corresponds to the time when the IMU was not working properly. Measurements made during this period (indicated by orange squares and dashed R_rs_ spectra) were excluded from further analysis.

**Figure 4:**
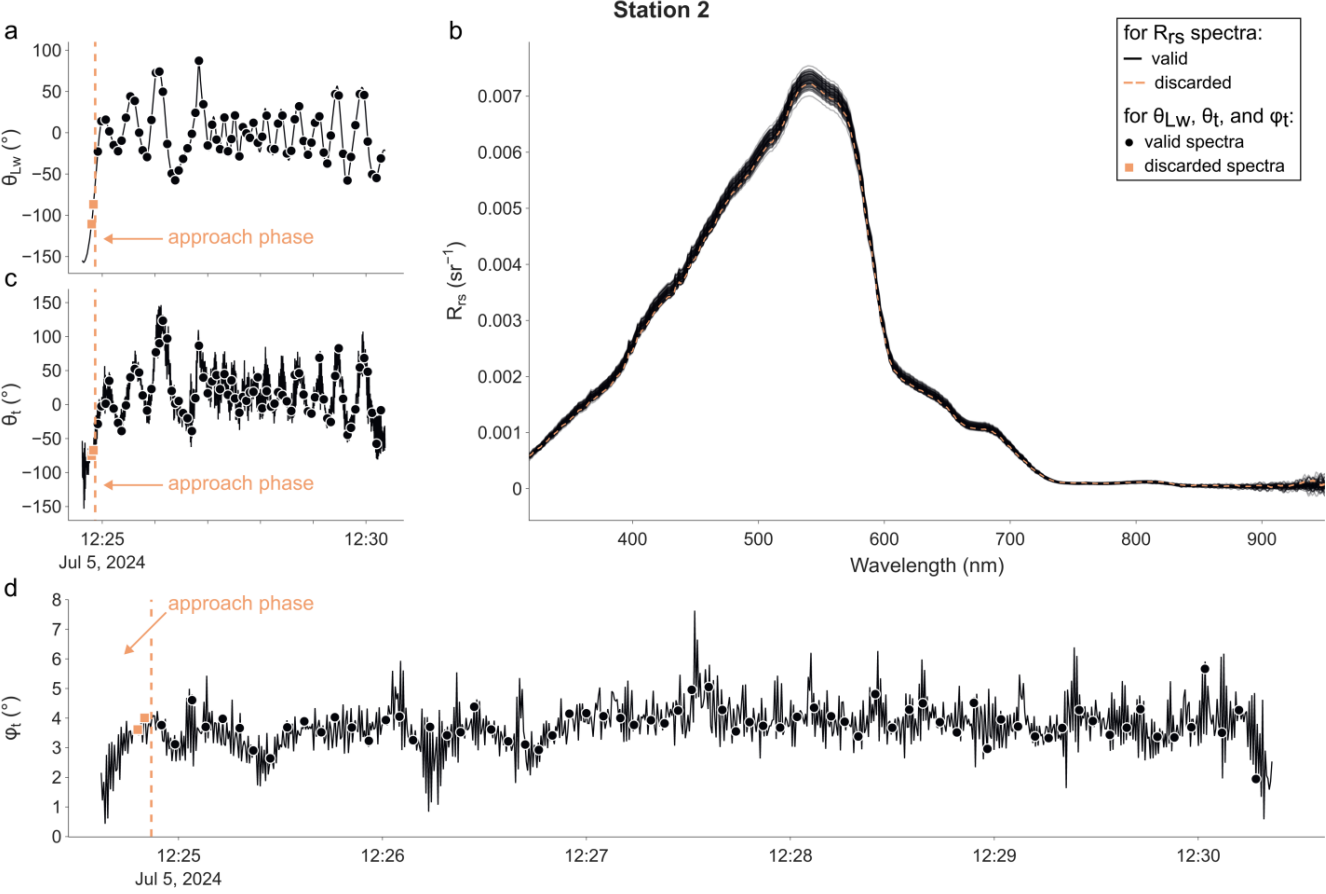
As in Figure 3, but for the 2nd station. The vertical dotted line (on panels (**a**,**c**,**d**)) shows the time at which the buoy started to be in position. Measurements made prior to this time (**b**) (indicated by orange squares and dashed R_rs_ spectra) were excluded from further analysis.

**Figure 5:**
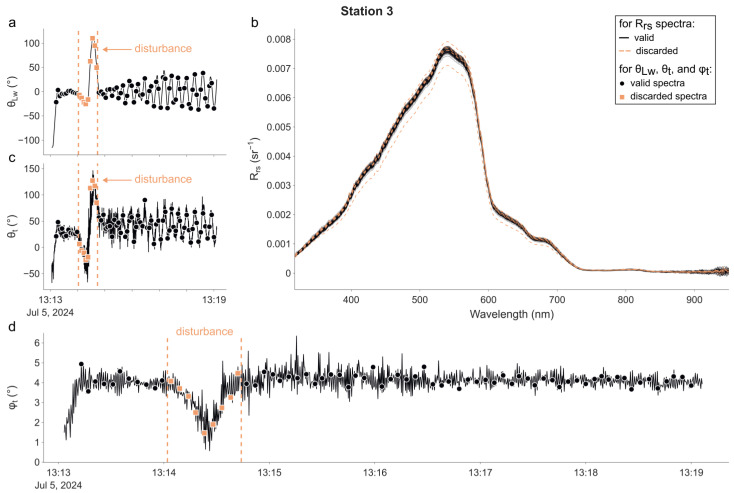
As in Figure 3, but for the 3rd station. The vertical dotted lines (on panels (**a**,**c**,**d**)) mark a period when the viewing geometry was not optimal. Measurements made during this period (**b**) (indicated by orange squares and dashed R_rs_ spectra) have been excluded from further analysis.

**Figure 6:**
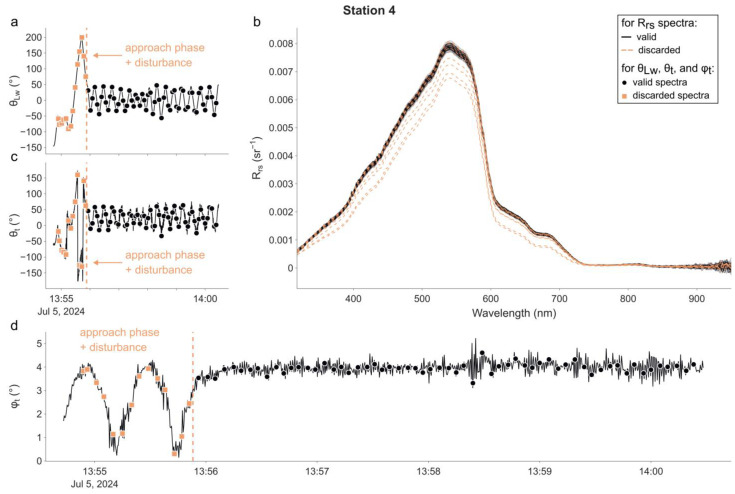
As in Figure 3, but for the 4th station. The vertical dotted line (on panels (**a**,**c**,**d**)) shows the time at which the buoy started to be in a stable position. Measurements made prior to this time (**b**) (indicated by orange squares and dashed R_rs_ spectra) have been excluded from further analysis.

The radiometric data are plotted in the same manner for each one of the four stations in Figure 3, Figure 4, Figure 5 and Figure 6. In each figure, the time series of θ_Lw_ is shown in panel (a), R_rs_ spectra in panel (b), time series of θ_t_ in panel (c), and φ_t_ in panel (d). The time at which the radiometric measurements were performed is represented for each time series using markers. Additionally, descriptive statistics and the distribution of θ_Lw_ (in total and per station) are available in the Supplementary Material (Figure S2 and Table S2). The campaign was performed without incident except at the 1st station, during which the magnetometer of the IMU was not working properly from 10 h30′23″ to 10 h33′41″. This led the radiance sensor to episodically face the wrong direction and thus measure a water volume in the direct shadow of the buoy. Therefore, we decided not to use any measurements made during this period in the following processing and calculation exercise.

For all stations, the measured R_rs_ spectra were typical of oligotrophic clear waters, with a maximum R_rs_ of approximately 0.0079 sr^−1^ at 540 nm. Slight absorption from CDOM is observed, but there is no chlorophyll-related spike at 670 nm. Thus, the R_rs_ spectra for wavelengths greater than ~800 nm are most likely sensor noise and will not be considered in further analyses. The water was very calm throughout the day—and even still for the 2nd, 3rd, and 4th stations—as illustrated by the recorded tilt of the buoy (Figure 3, Figure 4, Figure 5 and Figure 6d).

For the 1st station (Figure 3), θ_Lw_ started at −130° (−80° at the time of the 1st radiometric measurement) and rapidly converged to −0.21°, with an interquartile range (IQR) of 38.91°. The median of $\left|\theta_{Lw}\right|$ was 14.50°. θ_t_ appeared to be significantly correlated with θ_Lw_; thus, many spectra were measured when the tilt of the (ir)radiance sensor was directed towards the sun. The median of the tilt orientation θ_t_ was 3.05° (43.07° when considering $\left|\theta_{t}\right|$). φ_t_ was between 0° and 4°, and its evolution indicated the presence of small periodic waves during most of the sampling period.

For the 2nd station (Figure 4), θ_Lw_ started at −155° (−11° at the time of the 1st radiometric measurement) and rapidly converged to −6.15°, with an IQR of 40.8°. The median of $\left|\theta_{Lw}\right|$ was 20.72°. θ_t_ also looked correlated to θ_Lw_, and thus, many spectra were again measured when the tilt of the (ir)radiance sensor was heading towards the sun. The median of the tilt orientation θ_t_ was 11.20° (20.08° when considering $\left|\theta_{t}\right|$). However, given that the sun was at its zenith, the azimuth of the radiometers had little to no impact on the measurements. φ_t_ was between 0° and 7°, with a plateau at approximately 3.5–4°.

For the 3rd station (Figure 5), θ_Lw_ started at −115° (−22° at the time of the 1st radiometric measurement) and rapidly converged to −1.34°, with an IQR of 22.44°. The median of $\left|\theta_{Lw}\right|$ was 11.80°. Both θ_t_ and $\left|\theta_{t}\right|$ had a median of 38.66°. The amplitude of the tilt φ_t_ was approximately 4°. The short disturbance between 13 h14′04″ and 13 h14′42″ is due to the rope linking the buoy to our boat, which entangled itself with the light-blocking apparatus. It has been recorded in θ_Lw_, θ_t_, and φ_t_, as shown in Figure 5a,c,d. During this event, θ_t_ reached a minimum of −23.53° quickly followed by a maximum of 127.24°, and φ_t_ dipped to 1.46°. Nonetheless, the device rapidly converged to the ideal position in less than 20 s, demonstrating its quick capacity to recover from external perturbations.

For the 4th station (Figure 6), θ_Lw_ started at −146° (−59° at the time of the 1st radiometric measurement) and converged to 0.93° after 1 min, with an IQR of 47.94°. Measurements taken when the buoy was not yet in a stable position (i.e., left of the vertical green dotted line) were discarded for the radiometric performance assessment. The medians of $\left|\theta_{Lw}\right|$ and $\left|\theta_{t}\right|$ were 24.12° and 29.45°, respectively. φ_t_ ranged from 0° and 4° initially, with small oscillations created by boat-induced waves, and subsequently stabilised at a plateau of approximately 4°.

### 4.2. Comparison with Handheld SBA Measurements

In this section, we compared radiometric measurements from LSW $R_{rs}^{LSW}$—geometrically filtered, as explained in Section 4.1—to collocated simultaneous handheld SBA acquisitions $R_{rs}^{HH}$. As mentioned in Section 3.2, we also computed $R_{rs}^{LSW*}=L_{w}^{LSW}/E_{s}^{HH}$ so as not to be affected by calibration differences between the two irradiance sensors. The impact on the measurements of the aforementioned calibration differences is assessed in the following paragraphs.

**Figure 7:**
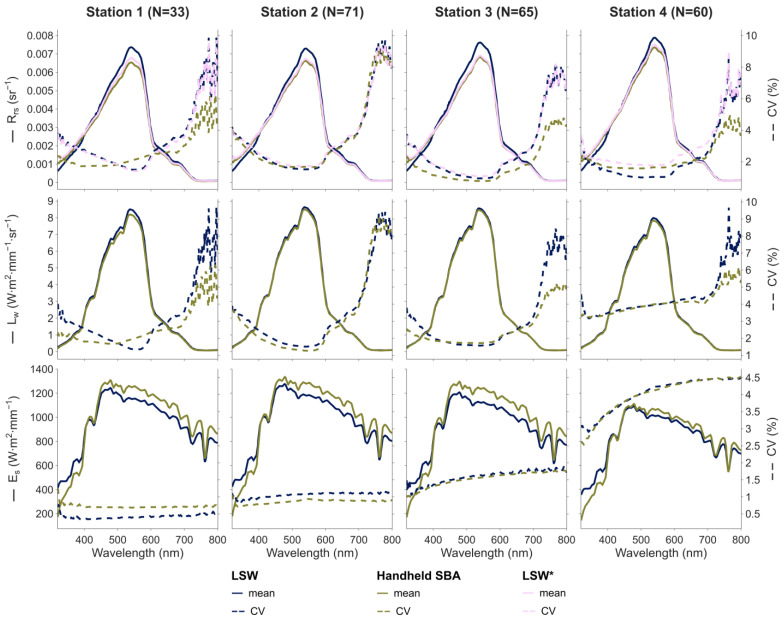
R_rs_/L_w_/E_s_ measurements at the four sampling stations in Bimont reservoir using LSW and the handheld SBA protocol. The mean spectra and CV are represented with solid and dashed lines, respectively.

The R_rs_, L_w_, and E_s_ spectra measured at each station are shown in Figure 7 along with their variability, evaluated by the coefficient of variation (CV). The dispersion was quite low for all protocols, with CV values for R_rs_ measurements below 4% in the visible spectrum (i.e., between 400 and 700 nm) and less than 10% for the whole spectrum (320–800 nm). The higher dispersion of L_w_ measurements at both spectral ends can be explained by the very low water reflectance at these wavelengths; therefore, small absolute variations arising from calibration discrepancies and sensor noise can result in large relative differences. The CV values for the E_s_ measurements were lower than 2% for the first three stations and slightly higher (<4.5%) at the 4th station, probably because of the presence of thin moving clouds. Overall, we observed that the CV values of both the L_w_ and E_s_ measurements were comparable (less than a few percent) between LSW and the handheld SBA protocol.

**Figure 8:**
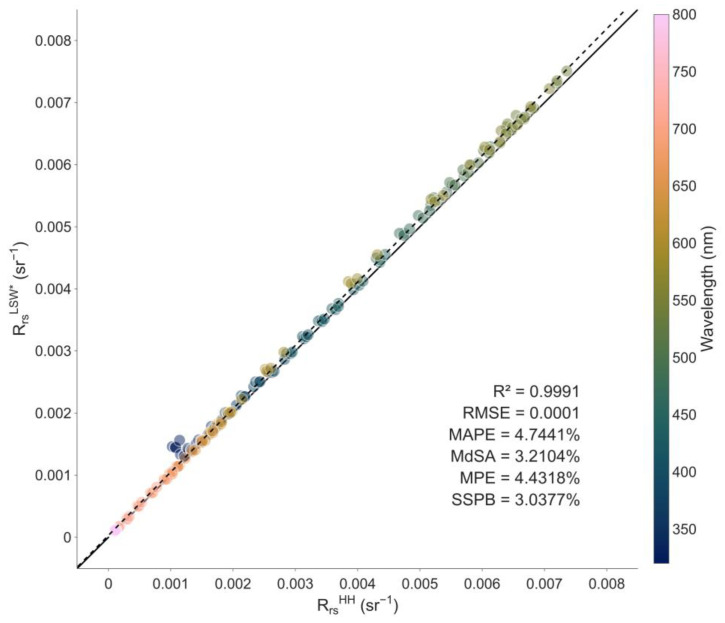
Comparison of R_rs_ measured by both LSW and the handheld SBA protocol in the 320–800 nm wavelength range. We used spectra with a 10 nm spectral step for the scatterplots, but statistics were computed with a spectral step of 1 nm. The solid and dashed lines represent the 1:1 and regression lines, respectively.

**Figure 9:**
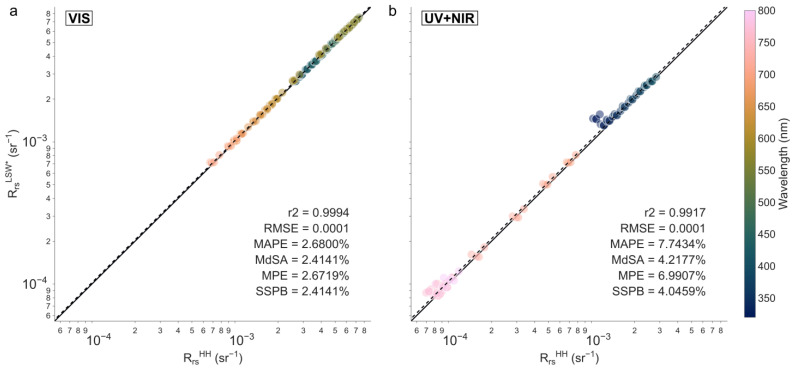
Comparison of R_rs_ measured by both LSW and the handheld SBA protocol. (**a**) Regression between $R_{rs}^{LSW*}$ and $R_{rs}^{HH}$ on the visible part of the spectrum (400–700 nm); (**b**) regression between $R_{rs}^{LSW*}$ and $R_{rs}^{HH}$ on the two spectral ends of the spectrum (320–400 nm and 700–800 nm). We used spectra with a 10 nm spectral step for the scatterplots, but statistics were computed with a spectral step of 1 nm. The solid and dashed lines represent the 1:1 and regression lines, respectively.

The R_rs_ spectra measured by both LSW and the handheld SBA display similar shapes but a slight bias (Figure 7 and Figure 8). Because both schemes provided consistent L_w_ spectra (Figure 7), this bias is due to the calibration offset between the two irradiance sensors (as mentioned earlier and illustrated in Figure S1), which explains the differences in the measured E_s_ spectra and their propagation to the R_rs_ spectra. Therefore, we used $R_{RS}^{LSW*}$ instead of $R_{RS}^{LSW}$ for the following performance assessment (Figure 8 and Figure 9).

Measurements from LSW and the handheld SBA system agreed well with each other: Figure 8 shows high correlation between $R_{RS}^{LSW*}$ and $R_{RS}^{HH}$ (r^2^ = 0.99) with a low bias and a high accuracy (SSPB = 3.0377% and MdSA = 3.2104%, respectively). However, performance in the visible and in the UV and near-infrared (NIR) parts of the spectrum warrants separate analysis. Bias and accuracy metrics (RMSE, MAPE, SSPB, and MdSA) across specific spectral regions are displayed in Figure 9. In the visible spectrum, the R_rs_ measurements from the two systems exhibited considerable similarity and fell below the targeted <5% accuracy threshold (MdSA < 2%). Conversely, in the UV and NIR regions, where R_rs_ values were substantially lower (see Figure 9), comparability was significantly diminished: we observed a two- to threefold increase in the different statistics. While the results obtained in the visible part of the spectrum are excellent in terms of stability and comparability, we want to highlight the difficulty in obtaining robust measurements of R_rs_ for wavelengths where L_w_ values are very small [32,33]; we advise future users to exercise caution when interpreting these spectral regions.

## 5. Discussion

### 5.1. LSW Radiometric Performances

The results of our experiment in Bimont reservoir demonstrated a high degree of concordance in R_rs_ measurements between LSW and the handheld SBA method, with a coefficient of determination > 0.99, a general accuracy (MdSA) of ~3.21%, and a bias (SSPB) of ~3.04%. These findings support the conclusion that LSW represents an effective implementation of the SBA protocol and constitutes a suitable alternative to handheld SBA measurements, offering enhanced autonomy and user-friendliness (as discussed in the following sections).

The R_rs_ measurement accuracy remained below the 5% threshold for the visible part of the spectrum (Figure 8), as intended to meet the highest level of standard requirements for water colour validation practices [34,35,36]. The lower accuracy at both ends of the spectrum was attributable to the very low L_w_ values of these oligotrophic waters.

Similarly, the dispersion was comparable for the two methods: below 4% in the visible portion of the spectrum (400–700 nm)—and even <3% between 400 and 625 nm—and up to 10% for wavelengths in the range of 700–800 nm. This is consistent with or even slightly better than previously published SBA measurements. For instance, Lee et al. [14] obtained in Lake Michigan and Green Bay a dispersion between 3% and 5% in the 350–600 nm wavelength range, 8% below 700 nm, and >15% for wavelengths greater than 780 nm. In Honghu Lake, Tian et al. [20] reported CVs < 4.5% in the visible spectrum and up to 13% at 750 nm. Finally, measurements carried out by Zibordi and Talone in the Western Black Sea [37] had a mean CV < 7% between 400 and 700 nm—and even <4% for wavelengths shorter than 600 nm.

Moreover, the minor discrepancies observed between the R_rs_ spectra acquired by the two systems can be explained by several factors. First, the radiometric calibration of the two radiance sensors was not conducted simultaneously, and despite their close correspondence, it is not perfect (Figure S1). The potential drift in the calibration coefficients of one or both sensors could contribute to the observed difference in the measurements, which is of a similar magnitude. Second, measurements were not taken at the exact same location, with handheld measurements being conducted closer to our boat, potentially introducing perturbations to the underwater light field.

### 5.2. Optimisation of the Viewing Geometry: The Motorised Rotating System

As demonstrated above, LSW has significant advantages for the accurate measurement of R_rs_. Using LSW, the duration of individual measurements can be significantly shortened. Indeed, as seen in Section 4.1 and discussed here, the rotating system of LSW allows the downward-looking radiometer measuring L_w_ to be successfully maintained in the direction of the sun throughout the acquisition sequence (the sun-relative azimuth distribution is shown in Figure S2 and Table S2). Consequently, we obtain more valid spectra (i.e., spectra acquired under optimal geometric conditions) in less time than using a standard non-motorised radiometric buoy as in [19]. The importance of not measuring a water volume in the shadow of the deployment structure is well illustrated in the literature [22], but also in this experiment. We can use the incident at the 1st station to illustrate this: the mean R_rs_ at 540 nm in shadow is approximately ~15% lower than when it is measured towards the sun (Figure 3). Therefore, it is important to determine the orientation of the radiance sensor for each measurement. This reinforces the pertinence of using an IMU onboard LSW to record essential metadata throughout the acquisition sequence: the absolute orientation of the radiometers with respect to the sun and its location. The latter facilitates the monitoring of potential buoy drift during measurements, which is also useful for selecting the appropriate pixel in satellite matchups if an image is simultaneously taken from space. The sun-relative azimuth of the radiance sensor θ_Lw_ can be derived from the absolute orientation of the buoy and is used to control the stepper motor (see Section 2.5). LSW absolute orientation is also used to derive LSW tilt: both its amplitude φ_t_ and its direction θ_t_. These three geometric values are crucial for the post-processing steps (or quality control) to eliminate or correct data that have not been acquired in optimal geometric conditions (e.g., due to waves or during the initialisation phase before its stabilisation in the optimal configuration; see Figure 6). The L_w_ spectra measured in the direct shadow of the buoy can be readily filtered using θ_Lw_. They can also be filtered using a threshold on φ_t_, as recommended in [19] ($\varphi_{t}<5{}^{\circ}$) and applied in [37] ($\varphi_{t}<3{}^{\circ}$). Finally, θ_t_ is important because a given φ_t_ will not have the same impact on the measured E_s_ if the buoy is tilted towards the sun or in the opposite direction. However, LSW does not yet offer a means of actively correcting its tilt as it does for the radiance sensor azimuth.

Given the importance of the IMU in acquiring this geometric information, the selection of a robust chip is highly recommended. The brief incident that occurred during the 1st acquisition sequence (as mentioned briefly in Section 4.1 and illustrated in Figure 3) highlighted that. The IMU onboard LSW (specifically its magnetometer) experienced an anomaly, manifesting as a ramp in its north reference. This resulted in the rotating system compensating for a non-existent rotation, consequently orienting the radiance sensor in a direction nearly opposite to that of the sun. Subsequent investigations revealed that this malfunction was due to the self-calibration feature of this particular IMU (BNO055 sensor; Bosh Sensortec GmbH, 72770 Reutlingen, Germany), which cannot be deactivated. While this does not invalidate the design of our prototype, it is advisable for future iterations of LSW to utilise alternative chips (e.g., BNO085/ICM-20948/LSM9DS1 or ISM330DHCX/LSM6DSOX/LSM6DS3TR-C + LIS3MDL).

As observed in Section 4.1 and Figure S2, the orientation control of LSW functions correctly with only basic instructions (see Section 2.5). The sun-relative azimuth angles of the radiance sensor at the time of acquisitions are centred at −1.34°, with Q1 and Q3 at −21.86° and 16.44°, respectively. The numbers would further improve with the exclusion of the 2nd station, where we were slowly moving to compensate for the drift we had during the 1st acquisition (see Figure 2), resulting in LSW being towed behind the boat. The performance of the orientation control system could be improved by fine-tuning the stepper motor parameters (the aimed velocity and its acceleration/deceleration). Its stability could be improved by refining the orientation control script, for instance by implementing a Kalman filter to predict the future orientation of the system. Such improvements should reduce the amplitude of the oscillation of θ_Lw_ around 0 (see Figure 3, Figure 4, Figure 5 and Figure 6a). Note that part of the θ_Lw_ dispersion also stems from the occasional rotation of the base (i.e., the float and the bottom half of the structure that is attached to it) instead of the payload. This phenomenon is attributable to multiple factors: (i) the circular shape of the float, (ii) the minimal fluid friction on the float, and (iii) the relatively low weight of the base relative to the payload. We attempted to address this issue by attaching a small (~29 × 21 cm^2^) PVC clippable fin to the device. While this modification mitigated the problem, the dimensions of the centreboard and its immersion depth of approximately 10 cm were insufficient.

Finally, because of the geometrical information collected by LSW during radiometric acquisitions, we observed that only retaining measurements performed when the sun-relative azimuth of the radiance sensor is <±20° (as carried out in [37]) may be excessively restrictive. Indeed, the spectra measured at a greater sun-relative azimuth during our experiment in Bimont reservoir did not appear to be affected by the shadow of the LSW structure. This is consistent with the modelling results presented in [22], which indicate that the azimuth effect was negligible when the heading of the SBA system was within ±120° of the ideal direction determined by the sun azimuth.

### 5.3. LSW Structure Advantages

In addition to recording ancillary geometric data that are useful for quality control, LSW addresses the main sources of uncertainty (other than self-shading) in field measurements via SBA. First, as mentioned in the previous section, its orientation system prevents the acquisition of L_w_ spectra in the direct shadow of the structure, which can occur with more conventional designs (as in [14,37]). This also allows LSW to be less susceptible to the effects of high solar zenith angles than alternative designs such as the radiometric buoy FOBY [20], as the radiance sensor is positioned between the float and the sun, rather than in the centre of the float (and thus potentially in its direct shadow). Second, the utilisation of an airtight screen significantly reduces the likelihood of fore-optics contamination, which occurs when the lens of the radiance sensor is submerged owing to wave action. Finally, the risk of the open end of the screen rising above the water surface is mitigated (i) by the carefully dimensioned attached screen and (ii) by the easy adjustment of the immersion depth of the screen and/or of the height of the radiance sensor, facilitated by the fixation clamp that secures it. In this study, the open end of the black screen was inserted below the water surface by 3.7 cm. Refs. [14,20] utilised depths of 5 cm and 2 cm below the surface, respectively. We did not characterise how the immersion depth of the screen affected SBA measurements, but since we employed the same radiometers (size and FOV) and screen for the two SBA protocols, it should impact them in a similar manner. Further experimentation would be necessary to quantitatively investigate the effect of the immersion depth of the screen on R_rs_ from SBA measurements under varying wind and wave conditions and water types.

Another important consideration was to ensure the user-friendliness of our device, which resulted in LSW being an easy-to-use solution for obtaining high-quality R_rs_ spectra. First, it allows for a significantly easier deployment compared with handheld SBA. Second, it is fully controllable via a smartphone over Wi-Fi using a straightforward yet powerful Python CLI, eliminating the need for a rugged laptop and obviating the use of long and often tangled cables. Furthermore, it does not require constant attention, and data are automatically acquired, enabling users to focus entirely on other tasks or measurements (e.g., water samplings or measurements of aerosol optical depth), which can represent a substantial time-saving and quality of life improvement. Additionally, in the case of the handheld SBA, maintaining the radiometer at the chosen immersion depth during the entire acquisition sequence can be challenging. Wave action or the movement of on-board operators can induce vessel tilt. Operator fatigue can also impact performance, particularly when utilising a sufficiently long boom to minimise interference from the boat. Furthermore, the tilt of the vertical pole (and, therefore, of the irradiance sensor) was not recorded and probably differed slightly from that on the hanging radiance sensor. While this can be an issue when collecting “real-world data”, it should not impact this comparison because we used the same E_s_ spectra/source for the two methods.

Due to all these ameliorations, high levels of accuracy were achieved with considerably less effort by simply deploying LSW onto the water surface, whereas we had to manoeuvre the boat throughout the measurement sequences to ensure that the radiance sensor used for the handheld SBA remained on the right side of the vessel.

In anticipation of its use for various field campaigns (including in remote locations), LSW has been made easy to transport. It is suitable for commercial air travel as it can be entirely disassembled, comprises small components, and does not contain lithium-based batteries. It also features a rapid separating mechanism that facilitates transport in the field, without requiring complete disassembly, and can be swiftly separated into two compact sections (1: the float and the bottom half of the structure and 2: the payload) using only four screws.

Furthermore, LSW is cost-effective, adaptable, and readily implementable. First, excluding the two radiometers, our device with its motorised orientation system costs just a few hundred euros. It is also significantly more economical than incorporating a second radiance sensor into a standard radiometric buoy, as proposed in [9], to avoid the measurement of L_w_ in the shadow of the device. Second, all the electronics, the stepper motor, slewing ring, waterproof container, etc., are open-source and/or are commercially available components. Additionally, the other parts are inexpensive as they are 3D-printed using affordable material and allow for adaptability to already owned sensors (provided they use Modbus RTU, a de facto standard transmission protocol) and any available components at the time of construction (for instance, any tube with sufficient buoyancy will suffice as a float). The height of the payload and of the radiometers is also freely adjustable using infinitely stackable wedges and/or the clamps that secure the sensors. It enables the use of different radiometers and/or protocols (e.g., above-water/contactless SBA; [38]), as well as the adjustment of the immersion depth of the screen. Finally, the structure comprises aluminium profiles—rendering it lightweight and robust—which are widely available and easily cut to the required dimensions. Assembly is achieved using widespread aluminium M4/M5 screws and nuts.

### 5.4. LSW Application in Ocean and Coastal Environments?

Despite its name, the use of LSW in coastal waters can be considered. Radiometric buoys implementing SBA have already been successfully used in coastal waters and open oceans [37,39]. However, operating such a device in a marine environment might be more complicated than in freshwater systems. First, the presence of larger waves would likely necessitate substantial data filtering during post-processing. It would also require the selection of a stepper motor with more torque (which implies larger batteries). Second, operating LSW in seawater would necessitate the use of a stepper suitable for saltwater submersion, which would slightly increase the cost of our device. We also expect an increase in both pitting and galvanic corrosion. Although the aluminium structure of LSW is anodised, and the two stainless-steel radiometers are already covered with rubber, minor galvanic corrosion may potentially occur between the aluminium screws and the stainless-steel clamps that maintain the radiometers. However, a protective coating can be applied, or, alternatively, the clamps can be made of plastic.

## 6. Conclusions and Perspectives

Our work is part of a broader effort by the international community to provide tools to enrich and catalogue the collection of accurate in situ measurements of water reflectance worldwide, thereby improving our knowledge of coastal and inland waters. This effort is reflected in the creation and maintenance of measurement networks such as AERONET-OC [34,40] and WATERHYPERNET [41], initiatives like SeaBASS [42], OC-CCI [43], and GLORIA [44], as well as the development of portable sensing solutions, which are necessary for studying remote or inaccessible water bodies. In line with the latter, LSW is a promising tool for obtaining high-quality water-leaving radiance measurements in lakes or any stagnant water bodies, offering a balance of accuracy, usability, and affordability that could benefit both research and monitoring applications in aquatic remote sensing. Our device addresses key challenges in “on-water” field radiometry through its motorised rotating system, which maintains the radiance sensor under optimal geometrical conditions (i.e., facing the sun). A comparison with handheld SBA measurements demonstrated excellent agreement, with a coefficient of determination >0.99 and a general accuracy (MdSA) of ~2.41% for remote sensing reflectance (R_rs_) in the visible spectrum (400–700 nm). LSW offers several advantages over existing SBA implementations:

- A shorter acquisition sequence due to automated sensor positioning (or a longer acquisition time with excellent stability);
- A reduced risk of fore-optics contamination;
- An improved ease of use and deployment;
- Cost-effectiveness and an adaptability to various sensors.

The system’s design allows for easy transport, rapid (dis)assembly, and straightforward operation via smartphone control. Its modular construction, using widely available components and custom 3D-printed parts, facilitates customisation and self-made repairs. While the prototype performed well overall, areas for potential improvement were identified, including refinement of the orientation control algorithm and addressing the occasional rotation of the float. Future studies should focus on optimising these aspects and conducting more extensive field trials across diverse water bodies and environmental conditions. Attaching both a full-sky and an above-water imaging camera is also under consideration. It would help to better characterise cloud distribution and hypothetical suboptimal measurement conditions, as well as observing what is being measured.

## Figures and Tables

**Table 1:** The three important angles to describe Lake SkyWater and its movement: θ_Lw_, θ_t_, and φ_t_. Both relative azimuths are clockwise positive (0° to 180°) and trigonometric negative (0 to −180°).

Symbol	Name	Value Range
θ_Lw_	sun-relative azimuth of the radiance sensor	(−180°, 180°]
θ_t_	sun-relative azimuth of the tilt (i.e., its direction, relative to SAA)	(−180°, 180°]
φ_t_	tilt (its “amplitude”)	[0°, 180°]

**Table 2:** List of all data measured by LSW, along with their sampling time, source (i.e., which sensor?), output file, and in which script they appear.

Value	Period	Source	Control Script	Output File
L_w_ (raw and calibrated spectra)	<4 s	radiance sensor	radiometric measurements	CSV file 1 (L_w_)
E_s_ (raw and calibrated spectra)	<4 s	irradiance sensor	radiometric measurements	CSV file 2 (E_s_)
time_1_	<4 s	RPi (data logger)	radiometric measurements	CSV files 1–2
integration time	<4 s	(ir)radiance sensor	radiometric measurements	CSV files 1–2
pre- and post-inclination	<4 s	(ir)radiance sensor	radiometric measurements	CSV files 1–2
latitude	1 min	GPS Bricklet	orientation	CSV file 3 (location)
longitude	1 min	GPS Bricklet	orientation	CSV file 3 (location)
time_1_	1 min	RPi (data logger)	orientation	CSV file 3 (location)
r	400 ms	IMU Bricklet	orientation	CSV file 4 (orientation)
time_2_	400 ms	RPi (data logger)	orientation	CSV file 4 orientation)

## Data Availability

The raw data supporting the conclusions of this article will be made available by the authors on request. The Python package for controlling the device, as well as the 3D models of all custom parts, are provided at https://github.com/inrae/Lake-SkyWater (accessed on 24 February 2025).

## Supplementary Materials

The following supporting information can be downloaded at https://www.mdpi.com/article/10.3390/s25051525/s1: Table S1: Power consumption of the embedded system; Figure S1: Radiometers intercomparison; Figure S2: Sun-relative azimuth distribution (per station); Table S2: Sun-relative azimuth distribution.

## References

[1] Yang H., Kong J., Hu H., Du Y., Gao M., Chen F. (2022). A Review of Remote Sensing for Water Quality Retrieval: Progress and Challenges. Remote Sens..

[2] Kuhn C., Butman D. (2021). Declining Greenness in Arctic-Boreal Lakes. Proc. Natl. Acad. Sci. USA.

[3] Free G., Bresciani M., Pinardi M., Giardino C., Alikas K., Kangro K., Rõõm E.-I., Vaičiūtė D., Bučas M., Tiškus E. (2021). Detecting Climate Driven Changes in Chlorophyll-a Using High Frequency Monitoring: The Impact of the 2019 European Heatwave in Three Contrasting Aquatic Systems. Sensors.

[4] Mishra D.R., Ogashawara I., Gitelson A.A. (2017). Bio-Optical Modelling and Remote Sensing of Inland Waters.

[5] Giardino C., Brando V.E., Gege P., Pinnel N., Hochberg E., Knaeps E., Reusen I., Doerffer R., Bresciani M., Braga F. (2019). Imaging Spectrometry of Inland and Coastal Waters: State of the Art, Achievements and Perspectives. Surv. Geophys..

[6] Lee Z., Carder K.L., Mobley C.D., Steward R.G., Patch J.S. (1999). Hyperspectral Remote Sensing for Shallow Waters: 2. Deriving Bottom Depths and Water Properties by Optimization. Appl. Opt..

[7] Borges H.D., Martinez J.-M., Harmel T., Cicerelli R.E., Olivetti D., Roig H.L. (2022). Continuous Monitoring of Suspended Particulate Matter in Tropical Inland Waters by High-Frequency, Above-Water Radiometry. Sensors.

[8] Zibordi G., Mélin F., Voss K.J., Johnson B.C., Franz B.A., Kwiatkowska E., Huot J.-P., Wang M., Antoine D. (2015). System Vicarious Calibration for Ocean Color Climate Change Applications: Requirements for in Situ Data. Remote Sens. Environ..

[9] Ruddick K.G., Voss K., Boss E., Castagna A., Frouin R., Gilerson A., Hieronymi M., Johnson B.C., Kuusk J., Lee Z. (2019). A Review of Protocols for Fiducial Reference Measurements of Water-Leaving Radiance for Validation of Satellite Remote-Sensing Data over Water. Remote Sens..

[10] Ruddick K.G., Voss K., Banks A.C., Boss E., Castagna A., Frouin R., Hieronymi M., Jamet C., Johnson B.C., Kuusk J. (2019). A Review of Protocols for Fiducial Reference Measurements of Downwelling Irradiance for the Validation of Satellite Remote Sensing Data over Water. Remote Sens..

[11] (2019). IOCCG Ocean Optics and Biogeochemistry Protocols for Satellite Ocean Colour Sensor Validation, Volume 3.0: Protocols for Satellite Ocean Colour Data Validation: In Situ Optical Radiometry.

[12] Ahn Y.-H., Ryu J.-H., Moon J.-E. (1999). Development of Redtide & Water Turbidity Algorithms Using Ocean Color Satellite.

[13] Tanaka A., Sasaki H., Ishizaka J. (2006). Alternative Measuring Method for Water-Leaving Radiance Using a Radiance Sensor with a Domed Cover. Opt. Express OE.

[14] Lee Z., Pahlevan N., Ahn Y.-H., Greb S., O’Donnell D. (2013). Robust Approach to Directly Measuring Water-Leaving Radiance in the Field. Appl. Opt..

[15] Mobley C.D. (1999). Estimation of the Remote-Sensing Reflectance from above-Surface Measurements. Appl. Opt..

[16] Lee Z., Ahn Y.-H., Mobley C., Arnone R. (2010). Removal of Surface-Reflected Light for the Measurement of Remote-Sensing Reflectance from an above-Surface Platform. Opt. Express.

[17] Kutser T., Vahtmäe E., Paavel B., Kauer T. (2013). Removing Glint Effects from Field Radiometry Data Measured in Optically Complex Coastal and Inland Waters. Remote Sens. Environ..

[18] Castagna A., Simis S., Dierssen H., Vanhellemont Q., Sabbe K., Vyverman W. (2020). Extending Landsat 8: Retrieval of an Orange Contra-Band for Inland Water Quality Applications. Remote Sens..

[19] Lee Z., Wei J., Shang Z., Garcia R., Dierssen H., Ishizaka J., Castagna A. (2019). On-Water Radiometry Measurements: Skylight-Blocked Approach and Data Processing. Append. Protoc. Satell. Ocean. Colour Data Valid. Situ Opt. Radiometry. IOCCG Ocean. Opt. Biogeochem. Protoc. Satell. Ocean. Colour Sens. Valid..

[20] Tian L., Li S., Li Y., Sun Z., Song Q., Zhao J. (2020). A Floating Optical Buoy (FOBY) for Direct Measurement of Water-Leaving Radiance Based on the Skylight-Blocked Approach (SBA): An Experiment in Honghu Lake, China. J. Geophys. Res. Ocean..

[21] Li Y., Tian L., Li W., Li J., Wei A., Li S., Tong R. (2020). Design and Experiments of a Water Color Remote Sensing-Oriented Unmanned Surface Vehicle. Sensors.

[22] Shang Z., Lee Z., Dong Q., Wei J. (2017). Self-Shading Associated with a Skylight-Blocked Approach System for the Measurement of Water-Leaving Radiance and Its Correction. Appl. Opt..

[23] Castagna A., Johnson B.C., Voss K., Dierssen H.M., Patrick H., Germer T.A., Sabbe K., Vyverman W. (2019). Uncertainty in Global Downwelling Plane Irradiance Estimates from Sintered Polytetrafluoroethylene Plaque Radiance Measurements. Appl. Opt..

[24] Tilstone G., Dall’Olmo G., Hieronymi M., Ruddick K., Beck M., Ligi M., Costa M., D’Alimonte D., Vellucci V., Vansteenwegen D. (2020). Field Intercomparison of Radiometer Measurements for Ocean Colour Validation. Remote Sens..

[25] Lin H., Lee Z., Lin G., Yu X. (2020). Experimental Evaluation of the Self-Shadow and Its Correction for on-Water Measurements of Water-Leaving Radiance. Appl. Opt..

[26] Morley S.K., Brito T.V., Welling D.T. (2018). Measures of Model Performance Based On the Log Accuracy Ratio. Space Weather.

[27] Pahlevan N., Mangin A., Balasubramanian S.V., Smith B., Alikas K., Arai K., Barbosa C., Bélanger S., Binding C., Bresciani M. (2021). ACIX-Aqua: A Global Assessment of Atmospheric Correction Methods for Landsat-8 and Sentinel-2 over Lakes, Rivers, and Coastal Waters. Remote Sens. Environ..

[28] Vansteenwegen D., Ruddick K., Cattrijsse A., Vanhellemont Q., Beck M. (2019). The Pan-and-Tilt Hyperspectral Radiometer System (PANTHYR) for Autonomous Satellite Validation Measurements—Prototype Design and Testing. Remote Sens..

[29] Zibordi G., Ruddick K., Ansko I., Moore G., Kratzer S., Icely S., Noorma A. (2012). In Situ Determination of the Remote Sensing Reflectance: An Inter-Comparison. Ocean Sci..

[30] Virtanen P., Gommers R., Oliphant T.E., Haberland M., Reddy T., Cournapeau D., Burovski E., Peterson P., Weckesser W., Bright J. (2020). SciPy 1.0: Fundamental Algorithms for Scientific Computing in Python. Nat. Methods.

[31] Moré J.J., Watson G.A. (1978). The Levenberg-Marquardt Algorithm: Implementation and Theory. Proceedings of the Numerical Analysis.

[32] Zibordi G., Hooker S.B., Berthon J.F., D’Alimonte D. (2002). Autonomous Above-Water Radiance Measurements from an Offshore Platform: A Field Assessment Experiment. J. Atmos. Ocean. Technol..

[33] Zibordi G., Melin F., Hooker S.B., D’Alimonte D., Holben B. (2004). An Autonomous Above-Water System for the Validation of Ocean Color Radiance Data. IEEE Trans. Geosci. Remote Sens..

[34] Zibordi G., Mélin F., Berthon J.-F., Holben B., Slutsker I., Giles D., D’Alimonte D., Vandemark D., Feng H., Schuster G. (2009). AERONET-OC: A Network for the Validation of Ocean Color Primary Products. J. Atmos. Ocean. Technol..

[35] Zibordi G., Berthon J.-F., Mélin F., D’Alimonte D., Kaitala S. (2009). Validation of Satellite Ocean Color Primary Products at Optically Complex Coastal Sites: Northern Adriatic Sea, Northern Baltic Proper and Gulf of Finland. Remote Sens. Environ..

[36] Hooker S.B., Esaias W.E. (1993). An Overview of the SeaWiFS Project. Eos Trans. Am. Geophys. Union.

[37] Zibordi G., Talone M. (2020). On the Equivalence of Near-Surface Methods to Determine the Water-Leaving Radiance. Opt. Express.

[38] Olszewski J., Sokolski M. (1990). Elimination of the Surface Background in Contactless Sea Investigations. Oceanologia.

[39] Wei J., Lee Z., Lewis M., Pahlevan N., Ondrusek M., Armstrong R. (2015). Radiance Transmittance Measured at the Ocean Surface. Opt. Express.

[40] Zibordi G., Holben B.N., Talone M., D’Alimonte D., Slutsker I., Giles D.M., Sorokin M.G. (2021). Advances in the Ocean Color Component of the Aerosol Robotic Network (AERONET-OC). J. Atmos. Ocean. Technol..

[41] Ruddick K.G., Brando V.E., Corizzi A., Dogliotti A.I., Doxaran D., Goyens C., Kuusk J., Vanhellemont Q., Vansteenwegen D., Bialek A. (2024). WATERHYPERNET: A Prototype Network of Automated in Situ Measurements of Hyperspectral Water Reflectance for Satellite Validation and Water Quality Monitoring. Front. Remote Sens..

[42] Werdell P.J., Bailey S., Fargion G., Pietras C., Knobelspiesse K., Feldman G., McClain C. (2003). Unique Data Repository Facilitates Ocean Color Satellite Validation. EoS Trans..

[43] Valente A., Sathyendranath S., Brotas V., Groom S., Grant M., Jackson T., Chuprin A., Taberner M., Airs R., Antoine D. (2022). A Compilation of Global Bio-Optical in Situ Data for Ocean Colour Satellite Applications—Version Three. Earth Syst. Sci. Data.

[44] Lehmann M.K., Gurlin D., Pahlevan N., Alikas K., Conroy T., Anstee J., Balasubramanian S.V., Barbosa C.C.F., Binding C., Bracher A. (2023). GLORIA—A Globally Representative Hyperspectral in Situ Dataset for Optical Sensing of Water Quality. Sci Data.

[45] Inkscape Contributors Inkscape. https://inkscape.org/.

[46] The GIMP Development Team GNU Image Manipulation Program (GIMP). https://www.gimp.org/.

[47] QGIS Association QGIS Geographic Information System. http://www.qgis.org/.

[48] Plotly Technologies Inc. Plotly Open Source Graphing Library for Python. https://plotly.com/python/.

[49] Crameri F. (2023). Scientific Colour Maps. Zenodo.

[50] Crameri F., Shephard G.E., Heron P.J. (2020). The Misuse of Colour in Science Communication. Nat. Commun..

