# Supplementary material for: Lake SkyWater—A Portable Buoy for Measuring Water-Leaving Radiance in Lakes Under Optimal Geometric Conditions
Source: Sensors (Basel). 2025 Feb 28;25(5):1525. doi: 10.3390/s25051525 (PMC11902623; doi:10.3390/s25051525)
Supplement: Supplementary file 1 [file sensors-25-01525-s001.zip › SM_LSW.pdf]

Supplementary Material

Quantity	Model	Power consumption	Current draw at 24V
1	Master Brick	410 mW	17 mA
1	Step-Down Power Supply		20 mA
1	Silent Stepper Brick		2 mA
1	GPS Bricklet 2.0		3 mA
1	IMU Bricklet 3.0	95 mW	4 mA
2	Isolator Bricklet	280 mW	12 mA
2	RS485 Bricklet 2.0	64 mW	3 mA
2	RAMSES G2	~1 W	42 mA
1	Stepper motor	≤ 75.84 W	< 1.58 A
1	Raspberry Pi 3B+	~1.8 W (idle)	75 mA
1	HAT Brick	100 mW	4 mA

Table S1. Power consumption for each element of LSW embedded system.

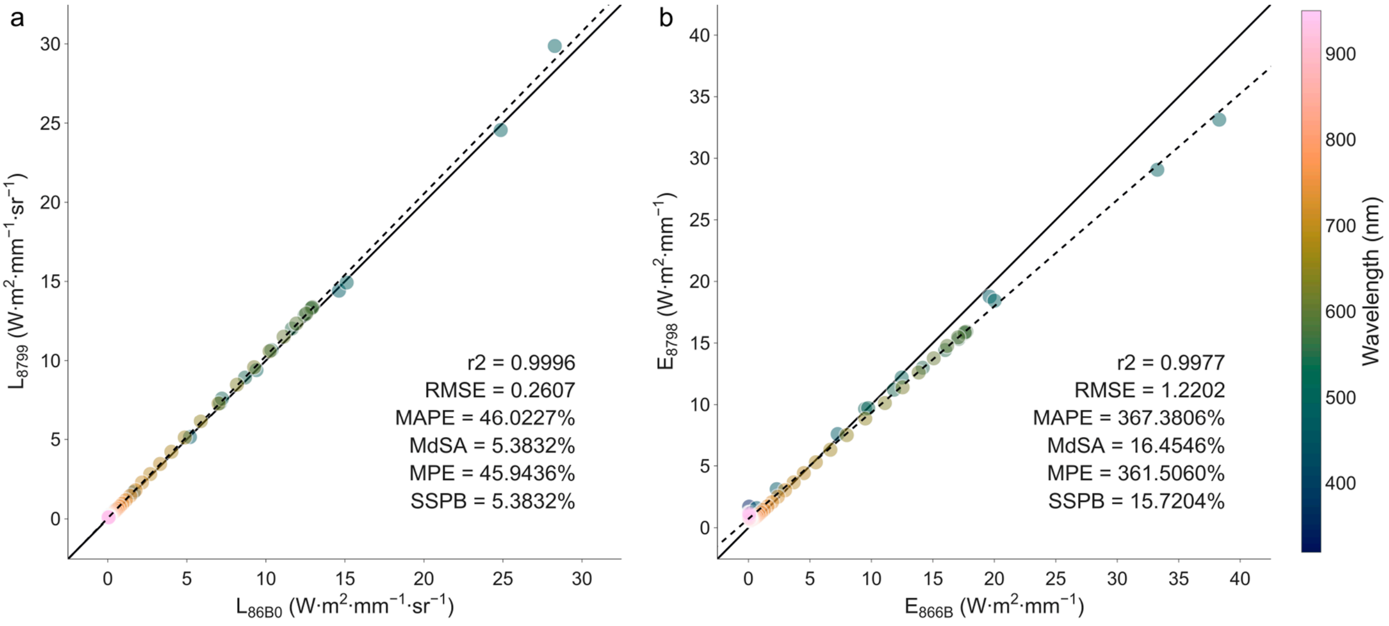

Figure S1. Comparison of the radiometers mounted on LSW (8799 and 8798) and those used for the handheld SBA protocol (86B0 and 866B) using a TriOS FieldCAL unit, for all wavelengths between 320 and 800 nm. **(a)** Regression between L_{8799} and L_{86B0}; **(b)** Regression between E_{8798} and E_{866B}. We used spectra with a 10-nm spectral step for the scatterplots, but statistics were computed with a spectral step of 1 nm. The solid and dashed lines represent the 1:1 and regression lines, respectively.

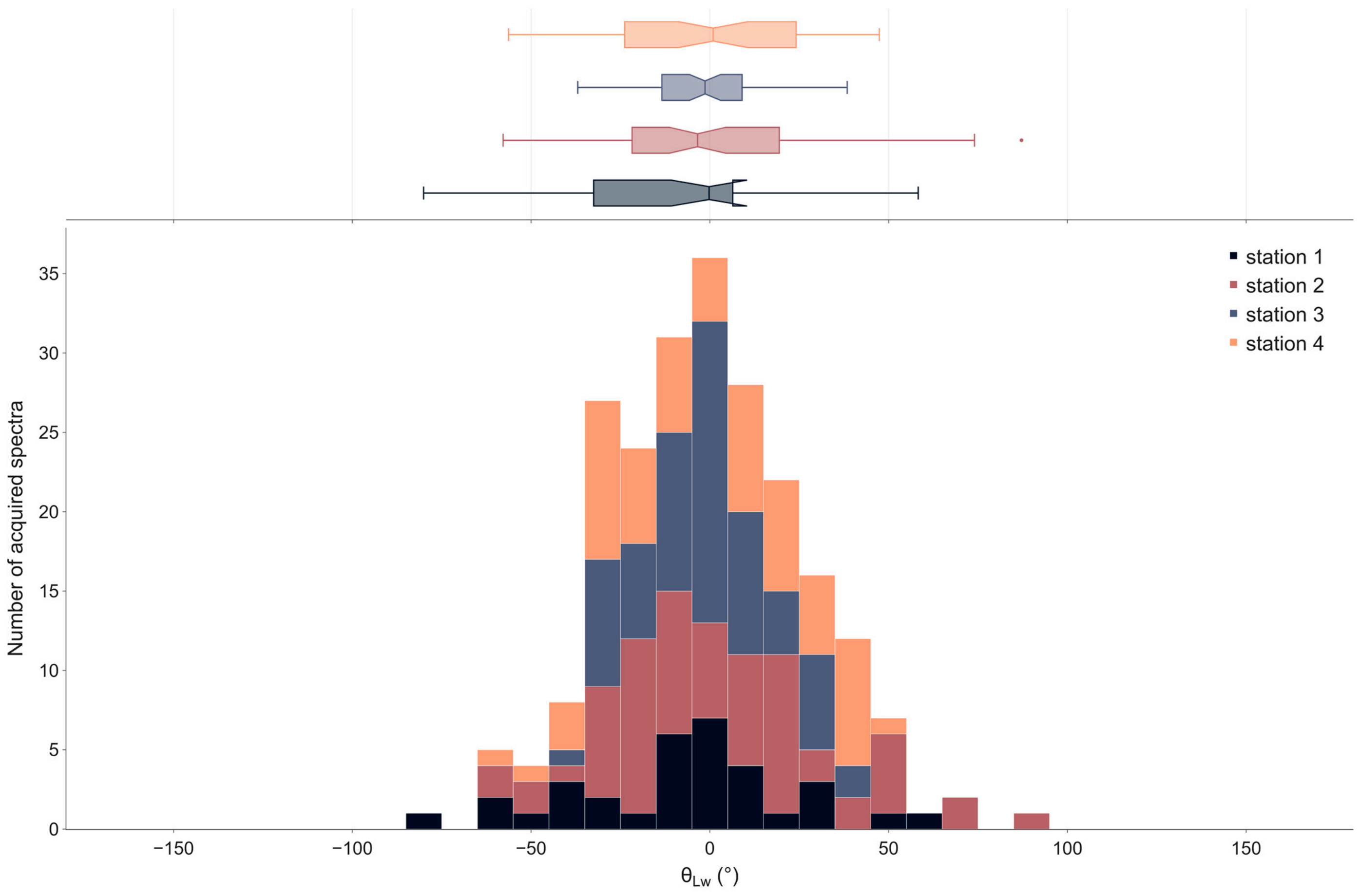

Figure S2. Sun-relative azimuth distribution (per station).

	Q1 (°)	Median (°)	Q3 (°)
Station 1 (n=33)	-32.50	-0.21	6.41
Station 2 (n=71)	-22.44	-6.15	18.34
Station 3 (n=65)	-13.43	-1.34	9.01
Station 4 (n=60)	-23.82	-0.93	24.12
**TOTAL (n=229)**	**-21.86**	**-1.34**	**16.44**

Table S2. Sun-relative azimuth distribution.
