# Supplementary figures and images for: Lake SkyWater—A Portable Buoy for Measuring Water-Leaving Radiance in Lakes Under Optimal Geometric Conditions
Source: Sensors (Basel). 2025 Feb 28;25(5):1525. doi: 10.3390/s25051525 (PMC11902623; doi:10.3390/s25051525)

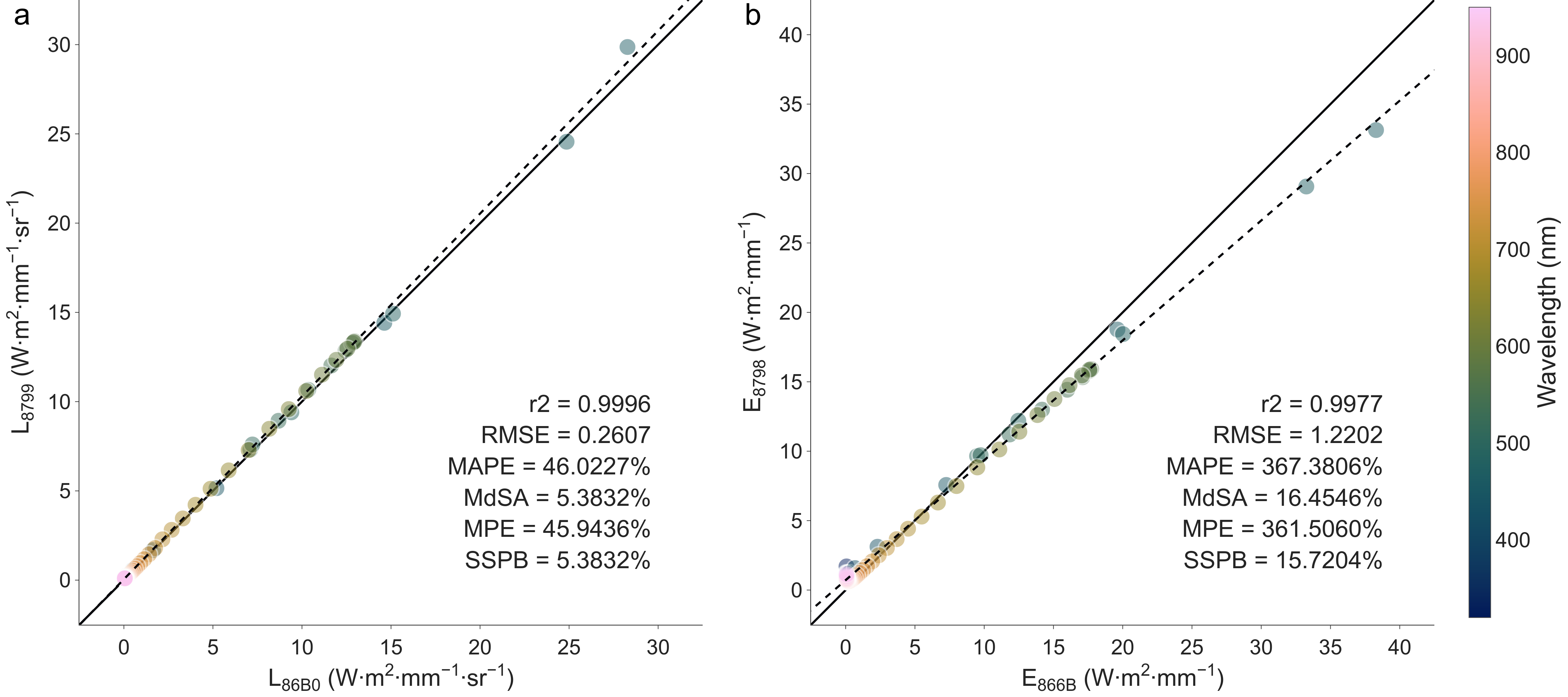

Supplement: Supplementary file 1 [file sensors-25-01525-s001.zip › figS1.png]

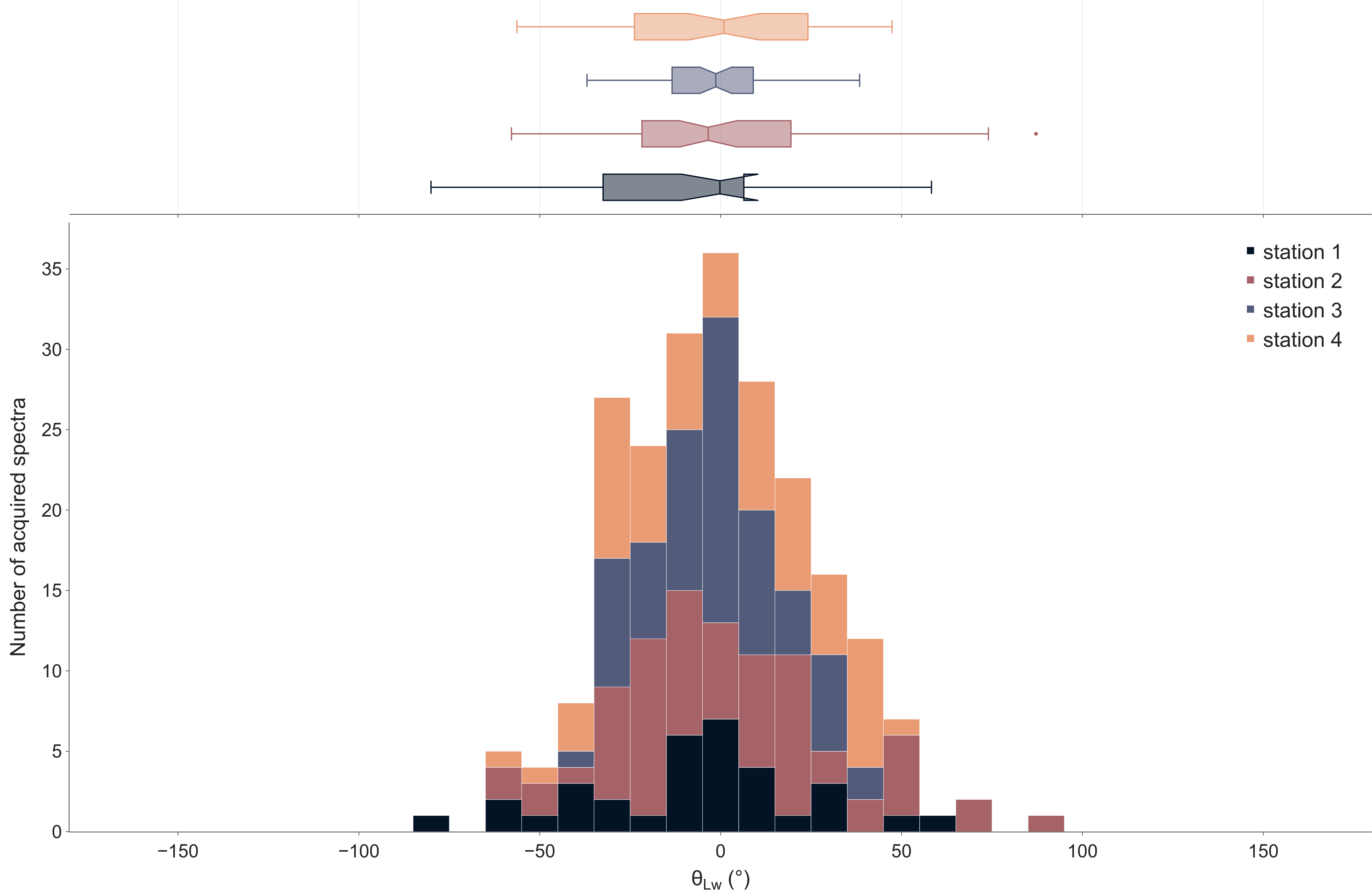

Supplement: Supplementary file 1 [file sensors-25-01525-s001.zip › figS2.png]
